# Obstructive Hydrocephalus Secondary to Cryptococcal Meningitis in an Immunocompetent Adult

**DOI:** 10.7759/cureus.18975

**Published:** 2021-10-22

**Authors:** Panduranga Seetahal-Maraj, Stanley Giddings, Kanterpersad Ramcharan, Narindra Ramnarine

**Affiliations:** 1 Neurosurgery, San Fernando General Hospital, San Fernando, TTO; 2 Faculty of Clinical Medical Sciences, The University of the West Indies, Trinidad, TTO; 3 Neurology, Surgimed Clinic, San Fernando, TTO

**Keywords:** csf diversion, immunocompetent, vp shunt, hydrocephalus, cryptococcal meningitis

## Abstract

Cryptococcal infections of the central nervous system (CNS) are common opportunistic infections in immunocompromised hosts. They can occur in immunocompetent hosts, and this has been documented in isolated case reports. Rapid neurological deterioration can be seen, particularly with hydrocephalus, and diagnosis can be difficult without a high index of suspicion. Treatment arms include prolonged antifungal therapy and cerebrospinal fluid (CSF) diversion procedures.

We present a case of a middle-aged immunocompetent male, who presented with an acute confusional state and papilledema. An urgent computed tomography (CT) and magnetic resonance imaging (MRI) revealed obstructive hydrocephalus, and an external ventricular drain was placed. CSF samples were collected, and analysis revealed cryptococcal infection. He was treated with antifungal therapy but failed external ventricular drain challenging. A ventriculoperitoneal shunt was placed after negative CSF studies were obtained.

While uncommon, cryptococcal meningitis in immunocompetent hosts can present with obstructive hydrocephalus. It can result in rapid neurological decline and death. Emergent CSF diversion and antifungal therapy are the primary treatment modalities. CSF diversion may be permanently required in some cases.

## Introduction

Cryptococcal infections of the central nervous system (CNS) are common opportunistic infections in immunocompromised hosts, particularly in the tropics [1]. It typically affects the respiratory tract and then disseminates hematogenously to the brain [2]. In rare instances, infection occurs in immunocompetent hosts, which has been documented in isolated case reports. Rapid neurological deterioration can be seen, particularly with hydrocephalus, and diagnosis can be difficult without a high index of suspicion [3]. Successful treatment may require both antifungal therapy and possible cerebrospinal fluid (CSF) diversion, including lumbar puncture, external ventricular drainage, or even ventriculoperitoneal shunting [4,5].

We discuss an immunocompetent patient who presented with signs of intracranial hypertension and hydrocephalus, with investigations revealing cryptococcal meningitis. The need for permanent CSF diversion needs to be tailored to each patient individually, and we report on our experience in managing one such case.

## Case presentation

A 57-year-old male presented to the emergency department with a progressively worsening confusional state and headaches for three days duration. His relatives noted that his memory had dramatically worsened over the previous 24 hours, and his consciousness was fluctuating. He had no fever, vomiting, or seizures. Past medical history was negative for immunosuppression or any significant comorbidities. He did not have a history of bird handling nor prolonged exposure to birds.

Physical examination revealed a lethargic appearing patient, with a Glasgow Coma Scale (GCS) of 13 (E3 M6 V4). His pupils were 4 mm bilaterally and sluggishly reactive to light. Cranial nerve examination was grossly intact, but papilledema was noted on fundoscopy. Motor and sensory examination of his limbs were normal aside from brisk reflexes (3+). He had no clonus and both plantars were downgoing.

**Figure 1:**
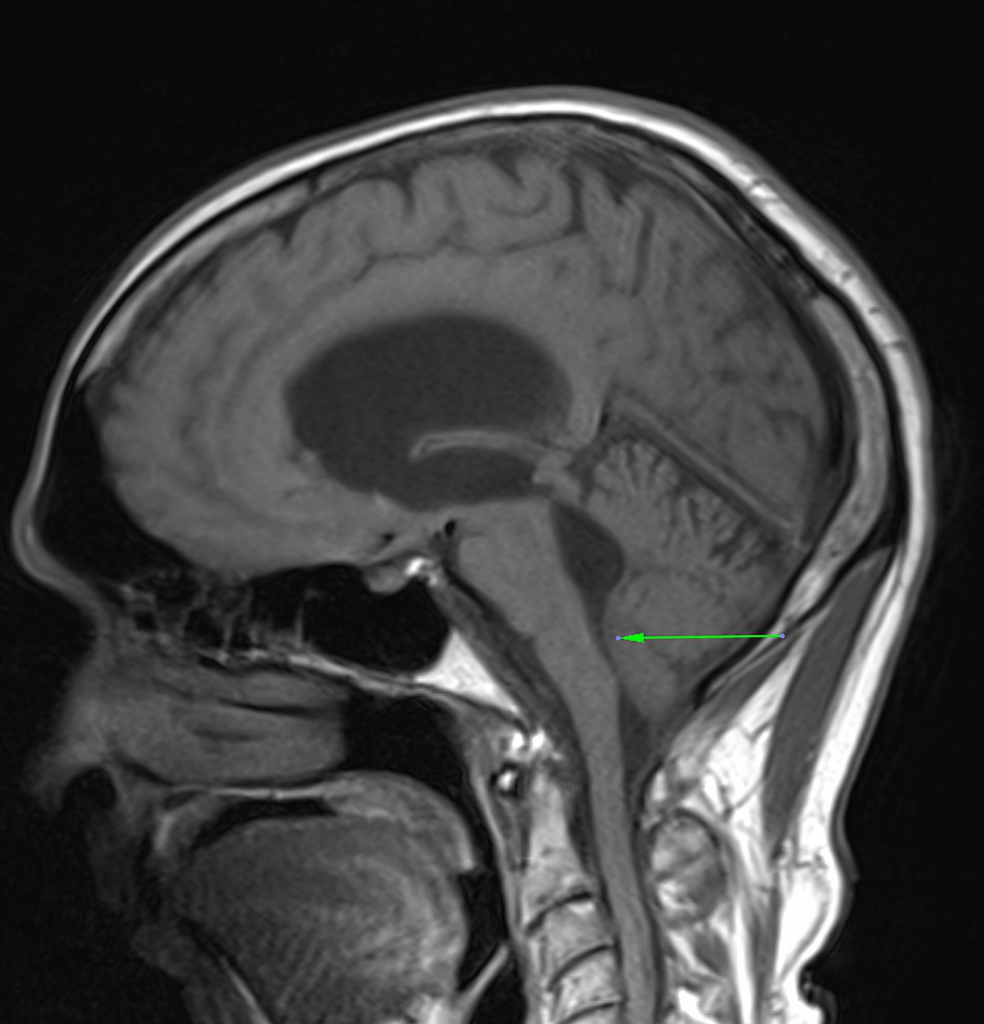
Sagittal T1-weighted MRI showing dilatation of the ventricular system, with stenosis at the floor of the fourth ventricle (arrow)

**Figure 2:**
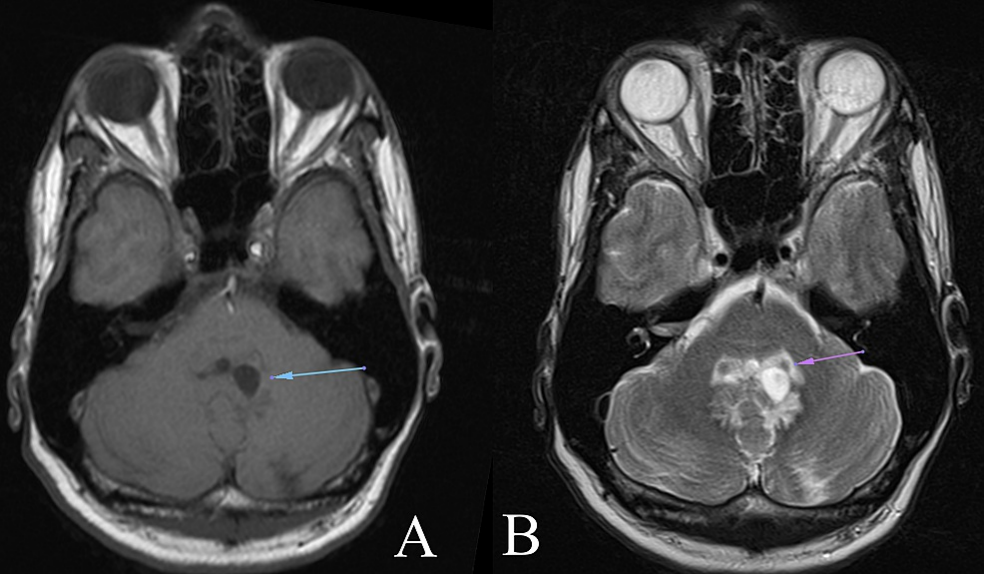
Axial T1 and T2-weighted MRI showing dilated foramina of Luschka (image A, arrow) and engorged choroid plexus (image B, arrow)

A brain computed tomography (CT) scan was done which revealed acute hydrocephalus. This was immediately followed by a magnetic resonance imaging (MRI) of the neuraxis, which revealed dilation of all ventricles and stenosis at the floor of the fourth ventricle (Figure 1). Hyperintensities in the periventricular regions on T2 and fluid-attenuated inversion recovery (FLAIR) sequences were noted, consistent with trans-ependymal CSF seepage. The choroid plexus was enlarged and hyperintense on T2-weighted sequences, consistent with choroid plexitis, with extension into the foramen of Luschka bilaterally. This obstructed the flow of CSF and led to dilation of the foramina of the Luschka and ventricular system (Figure 2). A nodule was also noted in the fourth ventricle, suspicious for a cryptococcoma. Unfortunately, no gadolinium was administered during this MRI scan.

The patient consented to immediate CSF diversion via an external ventricular drain, and CSF samples were sent for analysis. The patient had immediate improvement postoperatively, with the restoration of GCS to 15/15, and recovery of memory. His headaches ceased and there was no further papilledema. Postoperative CT showed resolution of hydrocephalus and good placement of the external ventricular drain (EVD).

CSF analysis revealed glucose of 23 mg/dL (normal 45-80), with a concurrent serum glucose of 102 mg/dL (normal 70-99). The CSF protein was 350 mg/dL (normal 20-40), and CSF white cell count was elevated at 480/mm^3^ (normal 0-8), with neutrophilia being predominant. PCR testing for cryptococcus neoformans was positive, consistent with cryptococcal ventriculitis. Quantitative cryptococcal antigen titres were not available in our setting. The patient’s serum white cell count was 14,000/ml (normal 4000-11,000), and his C-reactive protein (CRP) was elevated at 48 mg/L (normal 0-5). HIV testing was negative.

Antifungal therapy was commenced, with amphotericin 50 mg IV once daily and fluconazole 80 mg IV once daily. This was continued for four weeks until repeat CSF studies showed resolution of infection, with a negative PCR test for cryptococcus and normal parameters of glucose, protein, and white cell count. The EVD was kept in situ during this time and handled with an aseptic technique to avoid contamination. CSF studies were performed every 48 hours to ensure that no additional infection developed. After 14 days, repeat CSF studies were negative for cryptococcus, and the CSF glucose was 75 mg/dL (serum 99 mg/dL), CSF protein was 35 mg/dL and the CSF leucocytes were 2/mm^3^.

At this time, the EVD was challenged, by clamping it for a period of 24 hours and assessing the patient’s tolerance of this. After eight hours, he became disoriented to time and place, and a CT scan revealed hydrocephalus. This confirmed both clinical and radiological evidence of a lack of ventricular compliance. A decision was made for ventriculoperitoneal shunting as the definitive method of CSF diversion, and this was performed successfully. While endoscopic third ventriculostomy may have been an option, in this case, that procedure is not offered in our setting due to lack of equipment. The patient recovered neurologically and was discharged on long-term antifungal therapy with fluconazole for a one-year duration. At a three-month follow-up, he remains neurologically intact, with no evidence of intracranial hypertension.

## Discussion

The causes of hydrocephalus are multiple and varied, and fungal meningitis is an occasional etiological factor. Cryptococcal meningitis is an opportunistic infection that is known to primarily affect immunocompromised patients. It is the most common cause of meningoencephalitis in sub-Saharan Africa and Southeast Asia, with mortality rates ranging from 20% to 60% [1]. Up to 20% of these infections occur in the immunocompetent patient, with subsequent development of hydrocephalus [3]. This can result in an acute rise in intracranial pressure, resulting in permanent neurologic deficits or death. CSF diversion in the form of ventriculoperitoneal (VP) shunting was first utilized for this condition in the 1980s, however, it did not result in uniformly good outcomes. One series assessing factors that affected outcomes after shunting found that a GCS <8, and delay to surgery >48 hours were associated with a poor prognosis [6].

Pathophysiology

There are two main species of cryptococci that affect humans. *Cryptococcus neoformans* is responsible for the majority of infections and tends to cause more severe disease, whereas *C. gattii* is associated with a milder clinical course [7]. It is inhaled and enters the respiratory tract and haematogenously spreads to the CNS. Meningitis, encephalitis, choroid plexitis, and ependymitis are all sequelae of this. Anti-cryptococcal factors that are normally present in serum are absent in the CSF, and the polysaccharide capsule inhibits phagocytosis [8].

Hydrocephalus can arise from impaired CSF absorption and outflow when there is the involvement of the arachnoid villi. Fungal polysaccharide accumulates in these villous spaces, and further contribute to this [9]. Additionally, the presence of oedema, nodules, or cryptococcomas in the region of the fourth ventricle can also lead to obstructive hydrocephalus, as seen in our patient.

Intracranial hypertension due to cryptococcal meningitis has been recorded in both immunocompetent and immunodeficient patients. The exact mechanism of this is unclear, and one series by Cherian et al. did not discover any predisposing factors. Ventricular morphology was not related to the development of intracranial hypertension [4].

Clinical presentation

This is consistent with signs and symptoms of infection and intracranial hypertension. These may include fever, cognitive impairment, gait disturbance, headaches, vomiting, papilledema, seizures, and decreased consciousness [2,6]. It is not uncommon for patients to have chronic meningitis, and present in a delayed fashion.

Evaluation

Initial assessment should be with a non-contrast CT scan of the brain to confirm hydrocephalus. This should be followed by an MRI of the brain to determine whether the hydrocephalus is obstructive or communicating and if a lumbar puncture is contraindicated. Along with hydrocephalus, cryptococcomas, nodules, dilated perivascular spaces, pseudocysts, and enlarged choroid plexuses can be seen [8]. Confirmation of cryptococcal infection is typically done using CSF India ink preparation or cryptococcal antigen detection.

Management

There are two major arms of management - antifungal therapy and CSF diversion. Antifungal therapy is usually commenced with amphotericin B and fluconazole, for a minimum of two weeks. The goal is three consecutive negative CSF studies [5]. Oral fluconazole can then be continued for 12 weeks. Regarding CSF diversion in these patients, temporary measures such as trial lumbar punctures or EVD have been utilised. VP shunting has also been used with great success, as a permanent method of managing hydrocephalus due to cryptococcal meningitis, but the choice of procedure is not universally accepted. Endoscopic third ventriculostomy has been described as an alternative method for the management of obstructive hydrocephalus in this condition [3].

EVD has the advantage of the ease of CSF sampling and the capability to challenge the ventricles and avoid shunting. However, they may remain in situ for lengthy periods and cause superimposed ventriculitis, as well as be misplaced, malpositioned, and require repeated trips to the operating theater. Early VP shunting reduces these risks but may not guarantee a superior outcome [6,10]. The potential risk of seeding cryptococcal infection into the peritoneal cavity has not been definitively proven. The peritoneal cavity is thought to have less favorable conditions for cryptococcal growth, better antifungal penetration through the blood-peritoneum barrier, and more powerful phagocytes [11]. These factors are thought to greatly reduce the cryptococcal load post-shunting and improve the patient’s clinical condition.

Outcomes

The timing of CSF diversion is dependent on the patient’s symptomology. It was noted by Tang that patients who presented with acute signs and symptoms of hydrocephalus had better outcomes when shunted, compared to those who presented with symptoms for over a week’s duration [5,12]. Another study by Liliang et al. on 27 patients who were shunted for hydrocephalus in CM revealed poor outcomes when the GCS was <8 at the time of surgery. Over 80% of patients who had altered consciousness for >48 hours also had a poor response to VP shunting [6].

## Conclusions

Cryptococcal meningitis in immunocompetent patients remains a rare phenomenon. It can lead to obstructive hydrocephalus, intracranial hypertension, and acute neurological deterioration, resulting in death if not rapidly treated. CSF diversion in the form of serial lumbar punctures, external ventricular drainage, endoscopic third ventriculostomy, and VP shunting have all been shown to be successful in these patients. Further series with larger patient numbers need to be done, to assess the best management protocols for these patients.
